# Primary follicular lymphoma of the fallopian tube found incidentally in a patient treated for endometrial carcinoma: a case report

**DOI:** 10.1186/1746-1596-5-44

**Published:** 2010-06-28

**Authors:** Ahmad Alduaij, Katrine Hansen, Cunxian Zhang

**Affiliations:** 1 Department of Pathology and Laboratory Medicine, Rhode Island Hospital, APC 12 593 Eddy Street, Providence RI 02903 USA; 2 Department of Pathology and Laboratory Medicine, Women and Infants Hospital, 101 Dudley Street, Providence RI 02905 USA

## Abstract

We report a rare case of primary lymphoma of fallopian tube in a 68-year-old woman who underwent total hysterectomy and bilateral salpingo-oophorectomy for endometrial carcinoma. The specimen showed a well-differentiated endometrioid adenocarcinoma with superficial myometrial invasion. The left fallopian tube revealed a 1 cm nodule that histologically showed diffuse lymphoid follicles consisting of small cleaved lymphocytes and occasional larger cells. The cells were immunopositive for CD20, BCL-2, and BCL-6 but negative for CD3 and CD43. Polymerase chain reaction confirmed a monoclonal B-cell population. Fluorescence in-situ hybridization revealed at (14, 18) translocation. The patient had absent lymphadenopathy and negative CT scan of chest, abdomen, and pelvis. The findings were consistent with a primary low grade follicular lymphoma of fallopian tube. She did not receive chemotherapy and remained disease free for 13 months after surgery. Our case suggests that primary lymphoma of fallopian tube may be associated with a favorable prognosis.

## Background

Non-Hodgkin's lymphoma in the female genital tract is usually secondary, occurring as part of disseminated or systemic disease. The most common sites of involvement within the female genital tract are the ovaries and uterus. Lymphoma of the ovary extends to the fallopian tubes in about 25% of cases [1]. Primary lymphoma of the fallopian tubes is unusual, with only rare cases reported [2-4]. We now describe a primary follicular lymphoma of the fallopian tube, incidentally discovered during intraoperative consultation in a hysterectomy and bilateral salpingo-oophorectomy specimen performed for endometrial adenocarcinoma.

## Case presentation

A 68-year-old woman with a past medical history of hypertension, atrial fibrillation, and coronary artery stenosis presented with postmenopausal bleeding. Her gynecologic history included three spontaneous vaginal deliveries, normal periods every 28 days lasting 3 to 4 days, and menopause at age 50. She denied any history of sexually transmitted disease or hormone replacement therapy. Endometrial curettings showed a well-differentiated endometrioid adenocarcinoma associated with complex atypical hyperplasia. The patient had normal pre-operative laboratory tests including serum electrolytes, liver enzymes, and complete blood count. Her peripheral blood cells showed no significant morphological abnormalities. No palpable peripheral lymphadenopathy was noted. She underwent an exploratory laparotomy, total abdominal hysterectomy, and bilateral salpingo-oophorectomy. During intraoperative pathology consultation, we found an endometrial carcinoma with minimal myometrial invasion. The ampulla of the left fallopian tube showed a 1 cm nodule encasing the lumen. The nodule exhibited a white yellow and fleshy cut surface and was suspicious for malignancy. The question at the time of intraoperative consultation was whether this nodule represented metastatic carcinoma from the endometrium. A frozen section was performed and showed no evidence of carcinoma, but it displayed an atypical lymphoid proliferation. The left ovary and the right adnexa were within normal limits. Intraoperative exploration did not show evidence of extra uterine tumor or enlarged pelvic and periaortic lymph nodes. Based on these findings, surgery was concluded after total hysterectomy and bilateral salpingo-oophorectomy.

**Figure 1:**
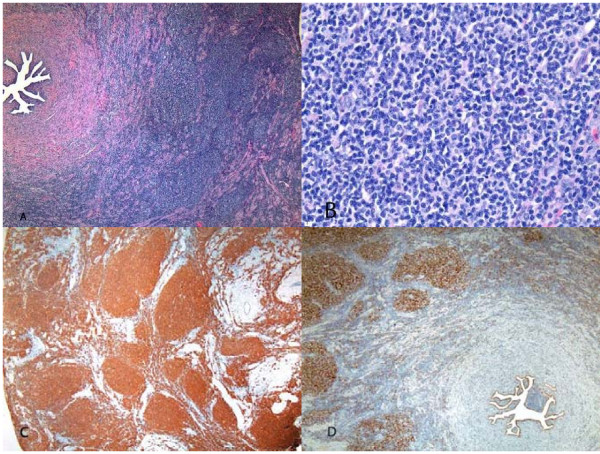
**Fallopian Tube Lymphoma**. Hematoxylin and eosin-stained section of the left fallopian tube showed diffuse back to back follicles without well defined mantle zones, involving the subserosa and outer muscularis but not the mucosa (A). At higher magnification, the follicles were composed of predominantly small cleaved lymphocytes admixed with rare large cleaved and large non-cleaved lymphocytes (B). The cells were strongly positive for CD20 (C) and BCL6 (D). Magnifications: 40× in A, C, and D; 400× in B.

Hematoxylin and eosin-stained permanent sections of the nodule in the left fallopian tube showed diffuse back to back follicles without well defined mantle zones (Figures 1A and 1B), involving the subserosa and outer muscularis but not the mucosa. The follicles were composed of predominantly small cleaved lymphocytes admixed with rare large cleaved and large non-cleaved lymphocytes. The cells displayed occasional mitotic figures, and the Ki67 immunostain showed scattered nuclear staining. The cells were strongly positive for CD20 (Figure 1C) but negative for CD3 and CD43, consistent with a monoclonal B-cell population. Bcl-2 and Bcl-6 (Figure 1D) immunostains highlighted the neoplastic follicles. Molecular studies using polymerase chain reaction technique confirmed a monoclonal B-cell population. Fluorescence in situ hybridization revealed at (14,18) translocation. Based on the histologic, immunohistochemical, molecular, and genetic findings, a diagnosis of low grade follicular lymphoma was made. There was no evidence of lymphoma in the rest of the specimen.

**Figure 2:**
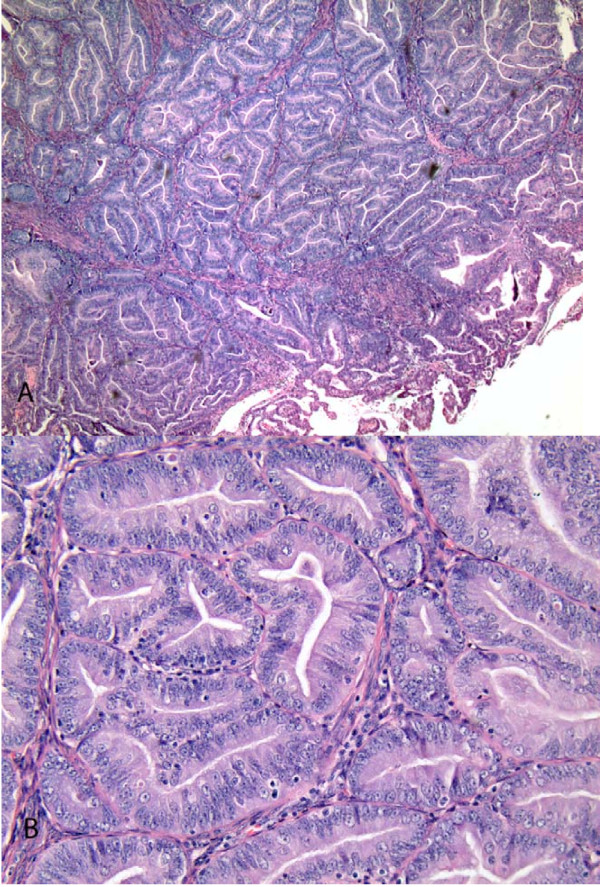
**Endometrioid Adenocarcinoma of the Endometrium**. At low magnification, the endometrial tumor showed a confluent glandular pattern in which individual glands merge to form a cribriform arrangement (A). at higher magnification, the confluent glands showed an absence of intervening stroma. The glands in this FIGO grade 1 tumor had round to oval nuclei with small nucleoli. The mitotic rate was low (B). Magnifications: 40× in A and 200× in B.

Permanent sections of the uterus showed a well differentiated endometrioid adenocarcinoma of the endometrium with less than one-third myometrial invasion (Figure 2A and 2B).

With absent lymphadenopathy and negative post-operative CT scan of the chest, abdomen, and pelvis, the lymphoma was considered a primary disease of the left fallopian tube and assigned stage IE according to the Ann Arbor staging system [5]. Considering that both the endometrial carcinoma and the tubal lymphoma were low grade and early stage, her doctors recommended close follow-up with radiologic imaging without chemotherapy. She was free of disease 13 months after surgery.

## Discussion

This was a rare case of primary tubal lymphoma incidentally discovered during surgery for endometrial carcinoma. At the time of surgery for endometrial carcinoma, surgeons often request an intraoperative pathology consultation for evaluation of disease extent that at least in part guides surgical management. Our patient showed a low grade endometrial adenocarcinoma with superficial myometrial invasion. With this finding alone, she would not need further staging surgery after hysterectomy and salpingo-oophorectomy. However, her left fallopian tube showed a small nodule suspicious for malignancy. Thus the intraoperative interpretation of this nodule became critical to the surgical management because a full staging procedure including pelvic lymph node sampling would be suggested if the nodule were metastatic endometrial carcinoma. Although carcinoma may show distinct gross features, accurate diagnosis relies on frozen section. In our case, frozen section of the left fallopian tube nodule displayed an atypical lymphoid proliferation instead of carcinoma, thus saving the patient from more surgery. The nodule on permanent sections proved to be a primary follicular lymphoma.

Although primary extranodal lymphoma is not infrequent, primary lymphoma of the fallopian tubes is very unusual. Only rare cases of primary tubal lymphoma have been reported. In one case, a 68 year old woman underwent hysterectomy and bilateral salpingo-oophorectomy for a tubo-ovarian mass and was found to harbor a primary follicular lymphoma of the tube, associated with hydrosalpinx and tubo-ovarian abscess [2]. Treatment and outcome information was not available for this patient. Another case was described in a 34 year old woman who presented with pelvic pain and an adnexal mass [3]. Pathologic examination of the excised adnexal mass revealed a primary tubal lymphoma of the mucosa associated lymphoid tissue type. The patient was disease free one year after surgery without chemotherapy. More recently, primary T-cell lymphoma of bilateral fallopian tubes was reported in a 51 year old woman who had presented with a pelvic mass [4]. She maintained remission for more than 5 years after treatment with chemotherapy. These cases show that primary tubal lymphoma, despite its rarity, can show various histologic subtypes including follicular center type, mucosa associated lymphoid tissue type, and T-cell lymphoma. We now describe the second case of primary follicular lymphoma of the fallopian tube. While the other patients in the literature presented with either pelvic pain or mass, the lymphoma in our patient was an incidental finding in a surgical procedure performed for endometrial carcinoma.

The etiology of primary tubal lymphoma is unclear. The association between chronic inflammation and certain types of extranodal lymphomas may have implications for lymphomas of the fallopian tube. Indeed, one previously reported case of primary tubal lymphoma showed associated hydrosalpinx and tubo-ovarian abscess [2]. It was, however, not clear whether tubal inflammation occurred before or after lymphoma in this patient. Because chronic salpingitis usually shows marked tubal distortion with hydrosalpinx, it is unlikely the cause of lymphoma in our patient who showed an otherwise normal appearing fallopian tube.

Lymphoma of the fallopian tube should be distinguished from other diseases. Carcinoma is usually easily distinguished from lymphoma because of the distinct histologic features between the two diseases. While carcinoma usually shows cellular cohesion, lymphoma often displays a diffuse pattern of noncohesive cells. Small cell neuroendocrine carcinomas may histologically simulate lymphoma, but nuclear molding, trabecular formation, and rosettes present in small cell carcinoma are absent in lymphoma. In difficult cases, immunostains for cytokeratin, CD45, synaptophysin, and chromogranin help make the correct diagnosis.

Lymphoma of the fallopian tube should also be distinguished from a chronic inflammatory process. While an inflammatory process shows a cellular heterogeneity, lymphoma usually displays a diffuse infiltration by a monomorphic population of lymphoid cells. Immunostains can help determine the clonality of lesional cells in difficult cases. Diagnostic challenges may arise in evaluating chlamydial salpingitis with a florid lymphofollicular hyperplasia mimicking follicular lymphoma [6]. Unlike follicular lymphoma that is positive for BCL-2, however, follicular hyperplasia is negative for BCL-2.

Accurate subtyping of lymphoma is imperative because various types differ in their rates of progression, overall prognosis, and response to individual forms of treatment. Although different lymphomas may show distinct histologic features, special studies play an important role in subtyping. The lymphoma in our case histologically displayed a follicular pattern consistent with follicular center type. BCL-2 and BCL-6 immunopositivity and t(14, 18) translocation confirmed a follicular lymphoma.

It is also important to distinguish a primary tubal lymphoma from secondary involvement. In general, distinction between primary and secondary lymphomas is facilitated by physical examination, radiologic imaging, and bone marrow biopsy. Since our patient did not show lymphadenopathy or evidence of lymphoma elsewhere, the tumor in the left fallopian tube was consistent with a primary tubal lymphoma. Our patient did not receive chemotherapy and remained disease free for more than 1 year.

## Conclusion

Primary fallopian tube lymphoma is rare, and reported cases indicate that various histologic subtypes can occur including MALT-type lymphoma, T cell lymphoma, and follicular lymphoma. Patients present with pelvic pain and/or a pelvic mass, but the lymphoma may be an incidental finding during pelvic surgery, as in our case. In this case it was important to distinguish this lymphoma from metastatic carcinoma at the time of intraoperative evaluation in order to spare the patient a staging procedure. The patient in this case did not receive chemotherapy and was disease free for more than one year suggesting a favorable biologic behavior associated with primary tubal follicular lymphoma.

## Competing interests

The authors declare that they have no competing interests.

## Consent

Written informed consent was obtained from the patient for publication of this case report and accompanying images. A copy of this written consent is available for review by the Editor-in-Chief of this journal.

## Authors' contributions

AA reviewed the fallopian tube lymphoma and confirmed this diagnosis. He also was a major contributor in writing the transcript. KH diagnosed and reviewed the endometrial adenocarcinoma, contributed to writing the transcript and is the corresponding author. CZ put the entire case in context, and contributed to the writing of the final transcript. All authors have read and approved the final manuscript.
